# Salutogenic Healthy Ageing Programme Embracement (SHAPE)- an upstream health resource intervention for older adults living alone and with their spouses only: complex intervention development and pilot randomized controlled trial

**DOI:** 10.1186/s12877-022-03605-3

**Published:** 2022-12-03

**Authors:** Betsy Seah, Geir Arild Espnes, Wee Tin Hong, Wenru Wang

**Affiliations:** 1 Alice Lee Centre for Nursing Studies, Yong Loo Lin School of Medicine, National University of Singapore, Clinical Research Centre, Block MD11, Level 3, 10 Medical Drive, Singapore, 117597 Singapore; 2 Health Concepts and Measurements-HealthierSG, 116B Rivervale Drive, #12-30, Singapore, 542116 Singapore; 3 NTNU Center for Health Promotion Research, Department of Public Health and Nursing, Faculty of Medicine and Health Sciences, Norwegian University of Science and Technology, Postbox 8905, Trondheim, N-7491 Norway

**Keywords:** Elderly, Intervention, Asset-based approach, Salutogenesis, Community care, Healthy ageing

## Abstract

**Background:**

In view of age-related health concerns and resource vulnerabilities challenging older adults to age in place, upstream health resource interventions can inform older adults about the availability, accessibility, and utility of resources and equip them with better coping behaviours to maintain health and independence. This paper described the development process and evaluated the feasibility of an upstream health resource intervention, titled Salutogenic Healthy Ageing Programme Embracement (SHAPE), for older adults living alone or with spouses only.

**Methods:**

A pilot randomised controlled trial design was adopted. SHAPE was designed to equip older adults with resource information and personal conviction to cope with stressors of healthy aging. This 12-week intervention comprised 12 weekly structured group sessions, at least two individual home visits and a resource book. Both the intervention and control groups received usual care provided in the community. Feasibility of SHAPE intervention was evaluated using recruitment rate, intervention adherence, data collection completion rate, satisfaction survey and post-intervention interview. Outcome measures (sense of coherence, health-promoting lifestyle behaviours, quality of life, self-efficacy, and self-rated health) were assessed at baseline and post-intervention. Paired t-tests were used to examine within-group changes in outcome measures. Content analysis was used to analysed qualitative data.

**Results:**

Thirty-four participants were recruited and randomised. While recruitment rate was low (8.9%), intervention adherence (93.75%) and data collection completion (100%) were high. Participants expressed high satisfaction towards SHAPE intervention and found it useful. Participants experienced mindset growth towards personal and ageing experiences, and they were more proactive in adopting healthful behaviours. Although the programme was tailored according to needs of older adults, it required refinement. Intention-to-treat analysis showed significant increase in overall health-promoting lifestyle behaviours, health responsibility, physical activity, spiritual growth, and stress management among intervention participants. However, they reported a significant drop in autonomy post-intervention.

**Conclusion:**

Findings of this pilot trial suggested that with protocol modifications, SHAPE can be a feasible and beneficial health resource intervention for older adults. Modifications on recruitment strategies, eligibility criteria, selection of outcome measures, training of resource facilitators and strong collaboration bonds with community partners would be needed to increase feasibility robustness and scientific rigor of this complex intervention.

**Trial registration:**

This study has been registered with clinicaltrials.gov on 10/05/2017. The trial registration number is NCT03147625.

## Background

Living arrangement has a major role in shaping the living circumstances, social environment, well-being, and the allocation of economic and care resources for older adults at old age. This socio-demographic characteristic has gained increased attention in recent years, as evident in the World Ageing Report [1] and World Population Ageing 2020 [2]. In the last decade, there is an increase in the number of senior-only households in Asia, including Singapore where older adults live either alone or with their spouses only [1, 3]. Decisions on living arrangements are influenced by personal preferences and choices, or circumstances beyond one’s control. These factors include older adults’ state of health, preference for autonomy, privacy and companionship, one’s ability to care for self and spouse, availability of age care support services, availability of kin for co-residence, willingness and ability of kin to provide care, and expectations of support by kin [4–7].

Older adults living alone or with their spouses only require resources such as personal financial assets, social capital, care support services and adequately funded healthcare. These enable them to live independently and care for themselves for as long as they can. These older adults also seek material, instrumental and emotional support from various formal and informal resources, i.e. family members, friends, neighbours, social workers, community services, home care, healthcare professionals, home-making, transport and emergency services [8–13]. They are dependent on others, yet do not want to be a burden; some had reservations for institutionalised care [8, 9, 14, 15]. As size of social networks decreases with age [16], older adults living alone or with their spouses only are socially vulnerable and at risk of social isolation. Past studies have reported that older adults with low levels of social activities and poor social networks/relationships were associated with poorer cognitive functions, functional decline, and higher readmission rates to hospitals [17–19]. In face of ageing and ailing health, the priorities of these older adults included having to stay healthy, safe, and independent in the comfort of their homes and community [6, 14, 20]. Despite the increasing need for formal and informal social support, studies reported that they experience insecurity and uncertainty due to the lack of, or difficulty accessing resources [12, 14]. When faced with daily caregiving challenges, some older couples from certain cultures or families had no kin to turn to and did not expect receiving aid from outside resources [21].

Singapore is a multi-ethnic Southeast-Asian developed country that shares the Confucian cultural heritage on filial piety. Therefore, its ageing policy focuses on ageing-in-place where the normative responsibility of senior caregiving first lies with the individual, followed by the family and government. In recent years, the Singapore government took a pro-active role in senior welfare provision, and channelled abundant resources into elderly-centric initiatives to make Singapore an age-friendly city [22]. However, most of these older adults were not highly educated and they might be less information savvy in keeping up with the development and usage of these resource initiatives. Some of them, especially those living alone or with their spouses only, have lesser familial exchange and support and might find it baffling navigating health knowledge and services within a resource-rich environment. To seek support, older adults navigate resources through existing relations. However, the engagement of such network-based support tends to be underutilised as it often depends on the proactivity of network members, or responses to life events [23]. In view of age-related health concerns and resource vulnerabilities challenging older adults to age healthily in place, upstream health resource interventions can inform the availability, accessibility, and utility of resources to equip older adults with better coping behaviours in maintaining health and independence for as long as possible. As such, a health resource intervention, titled “Salutogenic Healthy Ageing Programme Embracement (SHAPE)” was developed. This paper described the development process and evaluated the feasibility of the SHAPE pilot intervention among older adults living alone or those living with spouses only.

### Development of SHAPE- a healthy ageing health resource programme

The salutogenic model of health, which advocates for the creation of health using a stress-resource theory approach, was employed as an underpinning theoretical framework [24]. It offers a divergent perspective on how health can be managed through the salutogenic way of using resources to cope with stressors, as opposed to pathogenesis where attention is placed to unravel and mitigate the causes or risks of ill-health. The model has two core constructs: sense of coherence (SOC) and generalised resistance resources (GRRs). SOC characterises one’s worldview of life as having comprehensibility, manageability and meaningfulness [24], addressing the cognitive, behavioural, and motivational aspects respectively. GRRs are characteristics that facilitate individuals in coping with stressful situations, and they can be found within people as well as in their immediate and distant environments. SOC is developed through one’s engagement with and utilisation of GRRs [24]. Individuals with strong SOC are able to exercise flexibility and demonstrate adaptability in making the best health choices, by utilising GRRs to cope with difficult stressors or situations effectively, to maintain good health [25]. This theoretical framework was selected for its focus on resources, assets and strengths [26]. It musters positivity and rebuilds confidence among older adults to enhance their capacities and capabilities even in times of vicissitudes at old age. Moreover, the framework also recognizes the importance of creating and recreating life experiences which older adults accumulate over time, which influences their SOC through GRRs subsequently.

Healthy ageing lifestyle behaviours among elderly include physical activity, reduced sitting time, cognitive engagement, social engagement, sleep quality and sleeping habits, balanced nutritional intake and dietary habits, minimal or moderate alcohol consumption, abstinence from smoking and stress management [27–30]. However, many older community dwellers fell short of these recommended lifestyle behavioural goals [30, 31]. Considering that combined healthy lifestyle behaviours have positive cumulative effects on health outcomes and survival among older adults [28, 32], targeting multi-domain lifestyle behavioural modification is more beneficial as compared to single-domain lifestyle behaviour change. However, a systematic review reported that multi-domain health behaviour interventions are less effective among non-patient populations, as compared to patient populations due to lower motivation levels among the relatively healthy communities [33]. Therefore, intervention introduced to general community living older adults would need to be adequately motivating to make up for their ‘lower’ motivation. Other considerations on intervention delivery, such as the use of face-to-face presentation and expert facilitators (versus lay community facilitators), were reported to be more effective for multi-domain behavioural interventions [33]. As the effectiveness of any intervention is determined by the exposure and understanding of the programme [33], face-to-face interactions could increase the ease of implementation and receipt of a demanding multi-domain intervention while using expert facilitators could enhance recipients’ appreciation towards the intended behavioural change.

Self-efficacy is a prominent concept related to coping and health behavioural change. It refers to the belief of one’s abilities to successfully accomplish a task and produce desired effects [34]. Essentially, self-efficacy determines the initiation, effort, persistence, and achievement of behaviours. Previous studies have drawn similarities and established associations between SOC and self-efficacy, which influence health behaviours [24, 35, 36]. Conceptually, both SOC and self-efficacy act on the cognition, motivation and behaviour of an individual – having the belief to anticipate situations, perceive efforts invested being worthy of the valued outcomes, and manage the challenge ahead using perceived abilities and resources [24, 34, 37]. Empirically, positive and graded significant association was found between SOC and self-efficacy, with the strongest association observed in the lowest SOC [36]. Individuals with stronger SOC and higher self-efficacy scores exhibit greater positive health behaviours [38]. Self-efficacy was found to be a predictor of SOC among older adults, suggesting its position as a GRR [39]. Having the perceived capacity to act and achieve desired outcomes becomes a psychological resource that generates the behaviour to cope with the stressor faced.

**Fig. 1:**
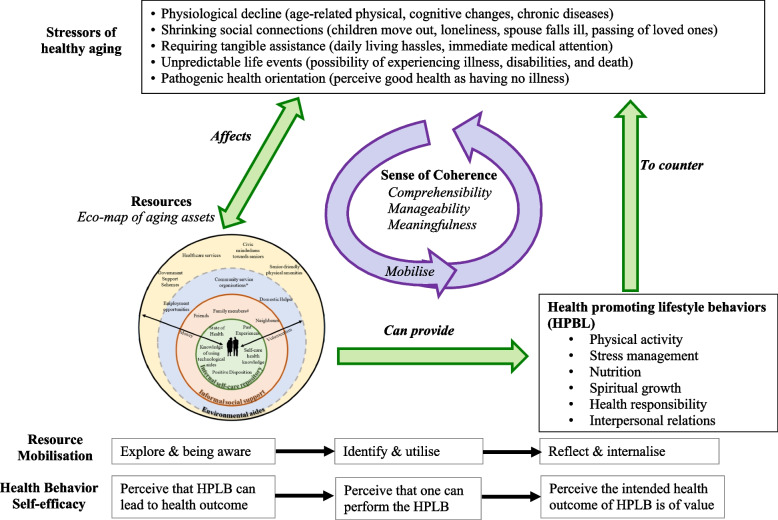
Conceptual framework of SHAPE intervention

To ensure the contents and approach of SHAPE intervention were contextually, socio-culturally appropriate, and theoretically grounded to the salutogenic model of health, we conducted a qualitative study among older adults living alone or those living with spouses only. Using focus group discussions, we explored their stressors of healthy ageing and the operationalisation of SOC in context of healthy ageing [40]. Ageing assets, which aided or can potentially aid to cope with stressors of healthy ageing, were also identified within the older adults and their external living spaces [41]. We developed the conceptual framework of SHAPE intervention (Fig. 1) based on the findings of the qualitative study.

The SHAPE intervention embraced the stressors of healthy ageing as salutary and facilitated older adults in identifying these stressors and understanding their coherence towards life at old age. To strengthen SOC, SHAPE aimed to (1) raise perceptual awareness towards and minimise the unpredictability of ageing-related experiences and daily living challenges (comprehensibility), (2) empower older adults to utilise their surrounding health assets to adopt health-promoting behaviours to cope with stressors and promote bio-psycho-social-spiritual health (manageability), and (3) activate older adults to seek motivation to live each day purposefully through individual reflection and making sense of old age experiences (meaningfulness) [40, 42]. Depending on the older adult’s circumstances and expectations, having an absence, shortage or mismatched fulfilment of an ageing asset, e.g., kinship and finances, can become a stressor that causes tension. As such, stressors and ageing assets share bi-directional relationships. As a health resource programme, SHAPE promoted the mobilisation of ageing assets via cognitive, behavioural and motivation pathways respectively: (a) explore and be aware of the function of their accrued resources, (b) identify, initiate and maintain the use of appropriate resource(s), in order to manage unique or situational stressors faced in everyday living, and (c) reflect and internalise the intent of using the resource(s), so as to appreciate the experience of engaging with the resource(s) [41]. Such an asset-based approach empowered older adults through learning and enhanced their internal capabilities in searching for solutions to develop health-promoting lifestyle behaviours (HPLB). Additionally, self-efficacy was mobilised through the cognitive, behavioural and motivation pathways respectively by encouraging older adults to (a) perceive HPLB can result in better health outcome, (b) perceive that they can perform the HPLB, and (c) the intended health outcome of HPLB is of value to them. Information on how the salutogenic model of health was further incorporated in the design of SHAPE intervention [42] and via salutogenic principles were detailed elsewhere [43].

## Methods

### Study design and study aims

We conducted a two-arm pilot randomized controlled trial study to evaluate the feasibility, acceptability, and potential effects of the SHAPE intervention. The following were the study aims:

1. To evaluate the feasibility of SHAPE intervention, pertaining to recruitment process, intervention adherence and outcome data, among older adults living alone or with spouses only.
2. To examine the acceptability towards SHAPE intervention including perceived satisfaction by older adults living alone or with their spouses only.
3. To examine potential effects of SHAPE intervention on SOC, self-efficacy, QoL, health promoting lifestyle behaviours and self-rated health among older adults living alone or with spouses only.

### Participants

Participants were recruited from a lower socio-economic and elderly-populated residential estate in Singapore. We partnered with the local community centre and recruited participants via convenience and snowballing sampling through various community engagement strategies such as flyer distribution at community events, poster advisement at residential areas and centres, word-of-mouth, and door-to-door canvassing. Eligible community dwelling older adults were aged ≥ 60 years old, either living alone or with another older adult and able to converse and read in Mandarin language. The age range of eligible older adults was initially ≥ 65 years old [42] and was later expanded to ≥ 60 years old to ease participant recruitment. Considering Chinese being the majority among the ethnic groups and the most common language literacy of Singapore’s elderly population, the pilot trial was conducted in Mandarin language. Older adults with self-reported diagnosis of dementia or mild cognitive impairment with Montreal Cognitive Assessment (MoCA) score < 20 [44], uncontrolled active psychiatric disorders e.g. schizophrenia, or moderate/severe depression with Geriatric Depression Scale (GDS) score > 10 [45], severe auditory or visual impairment and involvement in existing clinical trials were excluded. As this was a pilot study, the sample size was determined based on the pragmatics of recruitment and essentials for examining feasibility [46], adequate to pilot two rounds of intervention.

### Intervention

#### Salutogenic Healthy Ageing Programme Embracement

The SHAPE intervention was a multi-dimensional, person-centric, and asset-based health resource programme. The content of SHAPE intervention was designed to equip the older adults with resource information, the know-how resource utility, and the personal conviction in coping with stressors of healthy ageing (Table 1).

**Table 1:** Content of SHAPE intervention

**Stressors of Healthy Aging**	**Content of SHAPE intervention**
Decline in physiological functions	• Understanding natural biological aging process • Cognitive health and understanding dementia • Eating nutritiously • Engaging in functional physical exercises
Decrease in social network	• Coping with life transitions • Understanding & maintaining positive psycho-social-spiritual health
Circumstances requiring tangible assistance	• Understanding falls and maintaining home safety • Identifying common acute and chronic conditions, knowing when to seek assistance • Coping with illness and hospitalisation • Managing money and assets • Learning about end-of-life and planning for illness and death-related concerns
Unpredictable unfolding of negative life and health events	
Pathogenic health orientation at old age	• Understanding health from the salutogenic perspective • Appreciating aging as developmental process • Understanding own health needs • Living meaningfully

This 12-week SHAPE intervention adopted a mixed modal programmatic approach using 12 weekly structured group sessions, at least two individual home visits and a resource book.

Each 2.5-h group session covered on a specific topic, using a mixed use of didactic learning, classroom discussions and hands-on activities. During the break, healthy nutritious snacks were introduced, and practical information on where to purchase these snacks at economical prices or how to prepare them were also provided. Ten weekly 30-min physical activity sessions were incorporated to introduce exercises that promote older adults’ daily functional movements, e.g., reaching out for items from the top shelf or ground. At the end of each group session, participants were given homework to reflect upon and build on the topic introduced. As there is no specific recommendation on the group size of health education programmes, this pilot study strived to have 6 to 10 participants per intervention group class to facilitate sustained participation levels during group sessions. These group sessions were conducted at senior centres within the vicinity of participants’ residence, on either weekday afternoons or weekend mornings.

Home visits were core to the SHAPE intervention; they were personal reflective sessions to put together older adults’ existing and learnt resources into individuals’ context of life and focused on personal health concerns and specific resource needs. The first home visit explored and reflected upon the meaning of health and life at old age. The second home visit involved participants to evaluate their health and various aspects of life using an assessment toolkit comprising questions generated from the group session topics, followed by setting of personal health goals and developing action plans collaboratively with the resource facilitator. These health goals included, but not limited to, increasing frequency and intensity of specific exercises, attending regular health screenings, pursuing individual interests at leisure, discussing care preferences and will-making plans with family members. The SHAPE home visits recognised the heterogeneity of care needs among older adults and sought to enhance personal self-care skills according to individual needs.

The resource book was an integrative comprehensive health information book that complemented the contents of the group-based learning sessions and home visits. It was a compilation of seven wide-ranging self-care topics for daily living (e.g. ways to keep active, eating nutritiously, living at home safely, managing money and assets) and practical contextual information on the access and utility of local regional community facilities and services, and government schemes. Each topic was supplemented with information sources using QR codes to encourage interested older adults to cultivate information-seeking behaviour and deepen their resource knowledge.

**Figure:**
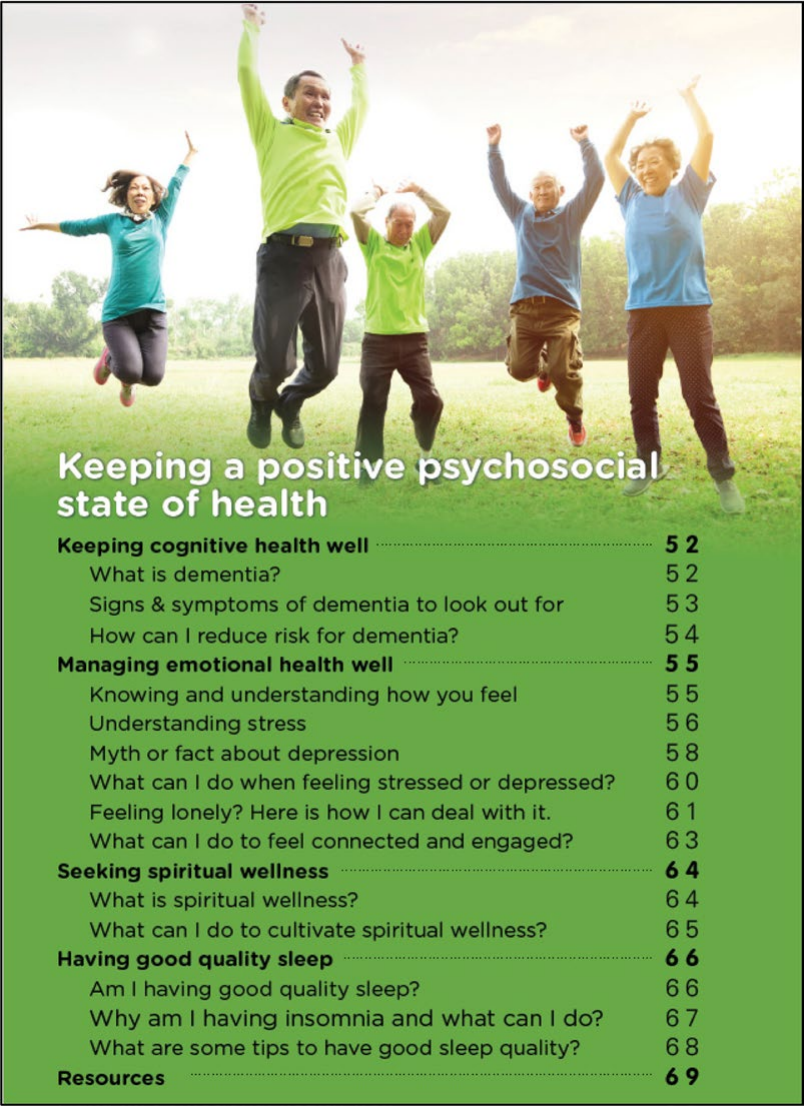
Images: Excerpts from SHAPE health resource book. All rights reserved.


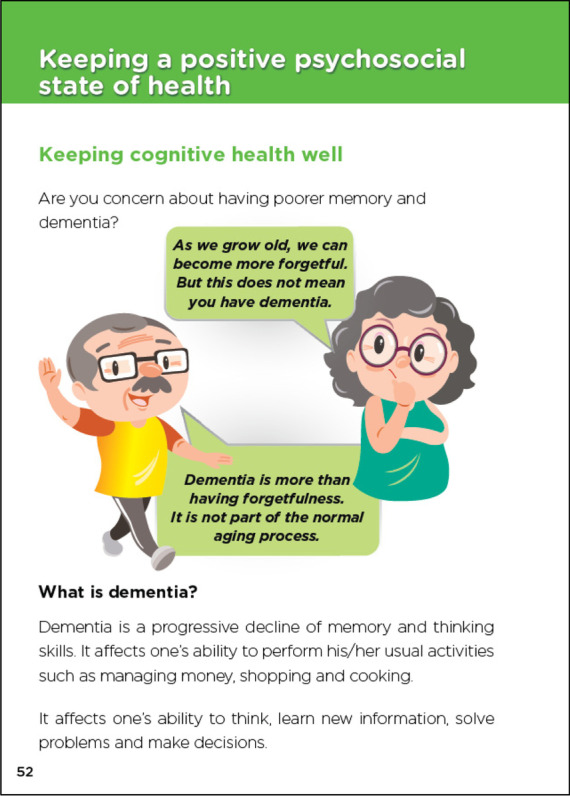


An intervention manual was developed to provide clear directives for the resource facilitator to conduct the group sessions and home visits. Both the intervention manual and resource book were content validated by a multi-disciplinary team of salutogenesis, geriatrics and gerontology experts. All group sessions and home visits were conducted by the first author, who is a registered nurse and developed the SHAPE intervention.

#### Usual care

Both the intervention and control groups received usual care provided in the community. All participants could continue to participate in the leisure activities offered at senior activity centres, community centres, non-profit organisations on their own accord. Examples of such activities included morning exercises, playing board/card games, art and craftwork, excursions to local attractions and festive celebrations.

### Randomisation

Eligible and consenting participants completed a baseline assessment prior to randomisation to either the SHAPE intervention or the control group. Randomisation was determined using computer-generated random number sequence and the group assignments were placed in sealed opaque sequentially numbered envelopes by the first author. To conceal random allocation sequence to screener prior to intervention assignment, a trained research assistant who performed the eligibility screening took the envelopes in numerical sequence and allowed participants to open the envelopes to reveal the group assignment. Blinding of group assignment to participants was least possible in this community-based study as they could mingle and share their group allocation with each other.

### Data collection

The same research assistant conducted the baseline and post-test quantitative data collection (after the conduct of SHAPE intervention at week 12) in the comfort of participants’ home or a nearby community centre. A separate post-intervention interview was conducted by the first author to understand the acceptability of intervention and she collect the qualitative data.

#### Outcomes for feasibility criteria

To establish trial viability and determine if changes in operation of main trial would be required, progression criteria on recruitment, intervention adherence and outcome data was set [47]. They were (1) 20% of older adults approached fulfilled the eligibility criteria, (2) 80% of participants randomised to intervention attended a minimum of eight group sessions (inclusive of 1^st^ week introductory and 12^th^ week consolidative sessions) and two home visits, (3) 80% of participants completed both pre-test and post-test data collection.

#### Acceptability of intervention

To determine the acceptability of SHAPE intervention, participants’ satisfaction was assessed using a self-developed 13-item evaluation form during a face-to-face interview post-intervention. Participants were asked to rate the overall programme and intervention components on group sessions, physical exercises, home visits and resource book on a five-point Likert scale from 1- strongly disagree to 5-strongly agree. This was followed by open-ended questions such as ‘what do you like the most about this programme?’, ‘what do you dislike the most about this programme?’, ‘what would you change to the programme to make it better?’ and a free-response question for participants to add other comments or suggestions about the programme and its components. As some participants were less proficient in writing, permission was sought to audio-record their verbal responses during the interview.

#### Outcome measures

Quantitative outcome measures collected for this pilot study include SOC, quality of life (QoL), self-efficacy, health promoting behaviours and self-rated health.

##### Sense of coherence

The 29-item Orientation to Life Questionnaire was developed to measure a person’s capacity in responding to stressful situations [24]. Using its short form questionnaire SOC-13, it measured 5 items on comprehensibility, 4 items on manageability and 4 items on meaningfulness. Each item was rated on a seven-point Likert scale and the total score ranged from 13 to 91, where higher scores indicate higher SOC. This cross culturally used instrument had been reported to be reliable and valid, with Cronbach’s alpha values ranging from 0.70 to 0.92 [37].

##### Quality of life

The World Health Organisation Quality of life Old module (WHOQoL-OLD) has been reported to be a valid and reliable cross-cultural geriatric-centric instrument [48]. This 24-item instrument consisted of six subscales: sensory abilities, autonomy, past, present and future, social participation, death and dying and intimacy. Each subscale had four items and the items were rated on a five-point Likert scale. Higher scores suggesting higher quality of life. The Chinese version of the instrument had an overall Cronbach’s alpha of 0.892 [49].

##### Self-efficacy

The Generalized Self-efficacy Scale (GSE) was developed to assess perceived self-efficacy with the intention to predict coping with daily events and adaptation of stressful life events [50]. It had ten items and they were rated on a four-point Likert scale. Examples of items include ‘I can always manage to solve difficult problems if I try hard enough,’ and ‘I am confident that I could deal efficiently with unexpected events.’ Good internal consistency of Cronbach α ranged from 0.76 to 0.90 had been reported [50], including the Chinese version [51].

##### Health promoting behaviours

The 52-item Health Promoting Lifestyle Profile-II (HPLP-II) measured multi-components of health promotion lifestyle behaviours. These six subscales are spiritual growth, interpersonal relations, nutrition, physical activity, health responsibility and stress management. The instrument was reported to have an overall internal consistency of Cronbach’s alpha 0.92, with its subscales alpha coefficients ranging from 0.70–0.91 [52].

Socio-demographic data including as age, gender, religion, marital status, education years, employment status, housing type and ownership, and brief medical history were collected.

### Data analysis

Feasibility outcomes were tabulated and reported descriptively and narratively. Quantitative data collected were analysed using the Statistical Package for the Social Sciences (SPSS) version 25. They were summarized using descriptive statistics, presenting results in raw count (%) for categorial data, mean and standard deviations for continuous variables. Chi-square and independent t-tests were used to determine difference for binary and continuous variables respectively between the intervention and control group. This pilot study was not powered to examine between group differences in outcome measures. However, paired t-tests were used to assess within-group mean differences between pre- and post- intervention outcome measures. Alpha was set at 0.05 for statistical significance.

Audio-recorded interviews were transcribed verbatim. Content analysis was used to analyse participants’ written responses and verbatim transcripts [53]. Codes were generated inductively from the meaning units in the raw data during decontextualization. This was followed by recontextualization where the researcher (BS) determined if each identified meaning unit addressed the aim. During the categorisation processes, extended meaning units were condensed and grouped into categories and themes. The themes, categories, sub-categories, and codes were reviewed and refined by two other researchers independently (WTH, WW) till consensuses were reached. Finally, data was compiled through report writing using meaning units to represent the studied phenomenon of interest.

## Results

**Fig. 2:**
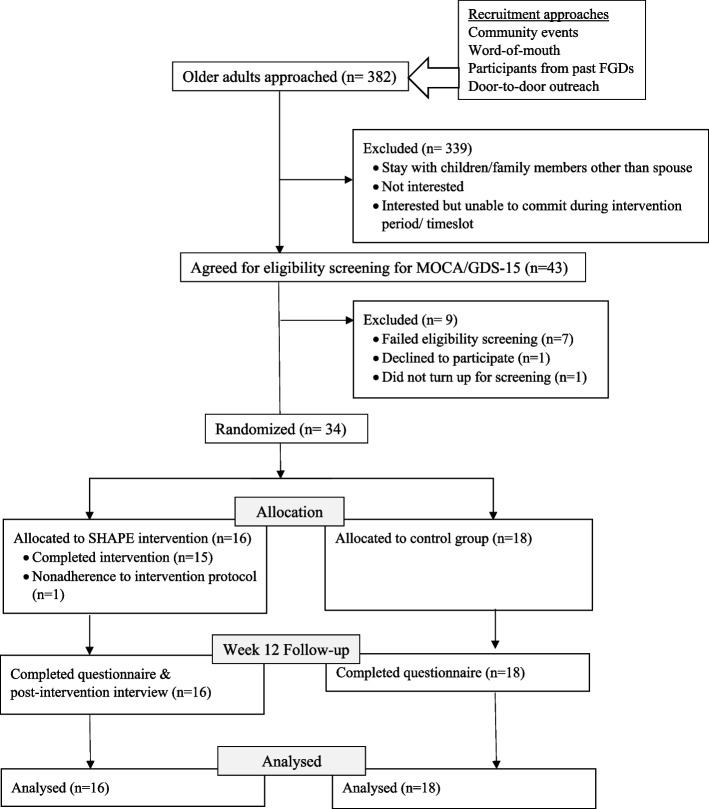
Flowchart on study recruitment and procedures

A study flow-chart is shown in Fig. 2. Among the 382 community-dwelling older adults approached, only 43 of them underwent the eligibility screening. 339 older adults were excluded because they were uninterested, unable to commit, or they reside with children/other family members. Those whom met the age and household eligibility criteria but declined to participate cited the following reasons: (1) they were old and did not see the need to attend health programmes, (2) attending classes would be stressful as they had little education, (3) the programme required weekly time commitment and they did not want to be bounded by that, (4) they could not retain information learnt and would be useless to attend the programme, (5) they did not want to mix with others during group sessions as ‘tongues will wag’, and (6) they had to take care of grandchildren on weekdays.

### Outcomes on feasibility criterion

Of those approached, 34 older adults enrolled into the study (8.9% recruitment rate) and were randomised to SHAPE intervention (*n* = 16) or control group (*n* = 18). All except one participant completed the SHAPE intervention (93.75% intervention adherence). The latter participant missed some group sessions to manage urgent family matters and she completed seven group sessions (inclusive of 1st and 12th sessions) and two home visits. All participants completed the questionnaire at baseline and follow-up at week 12 (100% outcome data).

#### Sample description

Participants’ social demographics and clinical data are presented in Table 2. Apart from the intervention participants being younger compared to the control participants, no significant differences in demographic characteristics, clinical data or outcome measures were observed between groups.

**Table 2:** Socio-demographic and clinical data of participants

Variable	Intervention group (*n* = 16)	Control group (*n* = 18)
**Age (Mean ± SD)**	70.00 ± 4.41	74.56 ± 5.42*
**Gender**		
Female	15	13
Male	1	5
**Religion**		
Buddhist	4	6
Catholic	1	0
Taoist	8	6
Christian	2	1
Others	1	5
**Years of education (Mean ± SD)**	6.25 ± 2.60	5.44 ± 3.59
**Education level**		
None	0	2
Primary	10	11
Secondary	6	4
Pre-U/University	0	1
**Employment Status**		
No former employment/ retired	9	13
Part-time	5	2
Housewife	2	3
**Marital Status**		
Single	3	1
Married	7	11
Divorced/Separated	3	0
Widowed	3	6
**No of children**	2.06 ± 1.34	2.39 ± 1.69
**Living Arrangement**		
Alone	8	6
Spouse/older family member	7	10
Unrelated person	1	2
**Housing Type**		
1 or 2-room	2	2
3-room	8	6
4-room	3	2
5-room	3	8
**House ownership**		
Self-owned	13	17
Family members/ rental	3	1
**Disposable income per month**		
< $500	3	6
$500-$750	5	4
$751-$1000	1	3
> $1000	7	5
**No. of medical conditions**	2.52 ± 2.31	2.56 ± 2.01
Hypertension	9	8
Hyperlipidemia	7	5
Diabetes	2	2
Heart Problems	0	2
Asthma	2	0
Osteoarthritis	6	6
Cancer	1	3
Kidney Problems	1	0
Stroke	1	1
Cataract	6	11
Depression	1	0
History of falls	8	5
**BMI**	23.58 ± 3.97	24.50 ± 3.91
**WHR**	0.90 ± 0.08	0.91 ± 0.77
**MOCA score**	26.63 ± 1 .50	25.33 ± 2.77
**GDS score**	2.19 ± 2.14	2.61 ± 2.10

*BMI* Body Mass Index, *WHR* Waist Hip Ratio, *MOCA* Montreal Cognitive Assessment, *GDS* Geriatric Depression Score

^*^P-value for t-test or Pearson Chi-square ≤ 0.05

### Acceptability of intervention

**Fig. 3:**
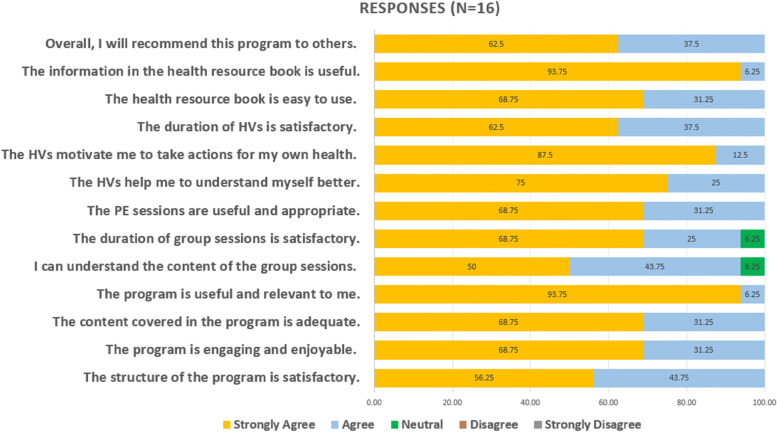
Bar chart on participants’ satisfaction towards the SHAPE intervention

Post-intervention evaluation of SHAPE provided by intervention participants were positive. They expressed satisfaction towards the overall of the programme and its components, with the majority reporting ‘agree’ or ‘strongly agree’ to the statements on the evaluation form (Fig. 3). Notably, 15 participants (93.75%) strongly agreed that the programme was useful and relevant, including the information found in the health resource book. The majority also strongly agreed that the home visits motivated them to take actions for their health (87.5%) and helped them to understand themselves better (75%).

Table 3 presents the themes and categories identified from participants’ open-ended responses. Two themes were identified: (1) usefulness of programme and (2) programme experience.

**Table 3:** Themes identified from participants’ open-ended responses on program evaluation

Themes	Categories	Sub-categories	Supporting quotes
Usefulness of programme	Mindset growth towards personal health and ageing experiences	Learning practical health knowledge about self-care in ageing	‘The program let us understand what to do, how to deal with situations in life and illnesses… how we would want to live our lives. For example, we need to face life situations encountered or when we were hospitalised. And to communicate with family, seek help from others, confide in friends, instead of handing these matters alone.’ (B4)
		Straightening out thoughts on personal ageing experiences	‘you interacted with peers such as older adults, you can listen and discuss about their experiences, views. They help to open up your mind. Otherwise, you feel lonely, no one can straighten your thoughts and you also do not know to manage these (aging) issues. Only after attending the class, you have solutions to manage them’ (A12)
	Being proactive and motivated to adopt healthful behaviours	Increase in self-care health practices	‘After joining SHAPE, I eat more nutritious food. Whenever I eat, I will think about the nutrition components… and I will scout and buy the foods. I signed up for a course on computer use at the community centre in the following month… because I want to learn something and … not to keep staying at home the whole day, need to go out and do some meaningful activities.’ (B9)
		Increase reception and proactiveness in health-seeking behaviours	‘There is a change in our health seeking behaviours, such as how to look for medical help, what (phone) numbers to call whenever emergency situation arises’ (B1)
		Experience physical and cognitive health benefits	‘the pushing against the wall exercises strengthen my arms. Also, we were taught how to sit on the floor and get up… Previously, I didn’t know how to sit and stand up properly and anyhow pressed onto both of my legs and had back pain.’ (A16)
Programme experience	Enjoyable positive learning experiences	A worthwhile programme	‘Time spent on the program is worthwhile…there was once when I had to go to (place) and could not attend. Apart from that, there are no other factors that I will not continue’ (B5)
		Satisfactory programme arrangement	‘Nothing in SHAPE I dislike. I like all activities, including the food and snacks introduced’ (A9)
		Positive interaction with instructor	‘The instructor is affable, sincere and develops relationships with participants… the long duration of time spent… facilitated communication.’ (A18)
	Suitability for seniors	Tailored for seniors	‘Programme is closely aligned to our lives, how to be better, how to change’ (A16)
		Catering for specific needs and preferences	‘Homework is not difficult, just that I am not able to write responses out.’ (A13) ‘I think some of the exercises might be vigorous for older adults.’ (B1)

#### Theme 1: Usefulness of programme

##### Mindset growth towards personal health and ageing experiences

Participants shared that they gained practical health knowledge about self-care and ageing. Not only did they learn about the ageing process and different types of illness faced at old age, but they also learnt how to respond to future health events, how to read food labels and eat healthily, how to ensure home safety and prevent falls, as well as to plan and manage their future care. Three participants mentioned that they shared the learnt health knowledge with their family members, friends, and neighbours.

‘The greatest advantage is to teach us on how to face and cope with living as older adults. Because we are of this age and do not have big plans or many thoughts. It helps us to understand how to face various events, face illness and death’ (B11)

Participants revealed that the programme content and the interaction with peers helped to straighten out their thoughts towards their personal ageing experiences. Few participants mentioned that content introduced was thought-provoking and the homework given made them reflect on their own health situations and future. Most participants expressed that their understanding and orientation towards life at old age changed. One participant realised that ‘even when you are old, you can have goals and pursuits’ (B5). Some were more aware of their life orientation after the programme and developed life goals which they strived to work towards. Others gained in self-confidence, were more responsible for own health or became ‘more at ease with self and have better control of our lives’ (B11). Overall, participants experienced mindset growth towards personal health and ageing experiences.

##### Being proactive and motivated to adopt healthful behaviours

Participants shared that they were more motivated to take actions for their own health after attending the programme. They shared how they paid more attention to self-care by increasing frequency of exercising, being more selective in consuming nutritious food, eating home-cooked food more often, spending more time with family and friends and signing up for classes to enrich themselves. Through the programme, some participants became more socially active and aware, and few mentioned that they ‘made new friends and would continue these friendship exchanges’(A9). A few participants also shared that they were in contemplation of engaging other healthful activities such as voluntary work.

Some participants also shared that they were more receptive and proactive in their health-seeking behaviors. Few mentioned that they ‘would read up more’ about health information (B1), others would be more prompt in seeking medical assistance and be proactive in attending health screenings. Nonetheless, few participants revealed that there was ‘no change in health-seeking behavior’ (B7).

‘I made a telephone call to arrange for (influenza & pneumococcal) vaccination tomorrow… Next, I will arrange for mammograph, and take the form from doctor and have my hearing checked out. So I have planned for them’ (B9)

Some participants fed back that they experienced improvements in their physical health after the programme. They experienced better mobility in walking and squatting, as well as lesser joint stiffness and aches. One participant claimed she became ‘leaner and lost 1 kg’ after practicing the introduced exercises (B4). A few participants added that their ability to write improved and had better memory after the programme.

‘The programme strengthens my muscles, and they will not ache or pain that much. (Now), I can move around more, not be afraid of going out. Whenever others asked me out, I no longer worry that I will not be able to walk. I am now more confident’ (B5)

#### Theme 2: Programme experience

##### Enjoyable positive learning experiences

Participants expressed that SHAPE intervention was a worthwhile programme which they found it enjoyable and beneficial. Content covered was comprehensible, more detailed compared to other health talks, and meaningful to them. Few of them indicated that it was a well-spent 12-week programme and would try not to miss any of the group sessions. Many mentioned that they would recommend the programme and looked forward to future learning experiences.

‘I saw the benefit of the programme. I told my family members I do not want to stay till Saturday for the (holiday) trip because I am interested to attend the programme. It is fun and interesting, so no choice my daughter had to fetch me back (home) earlier while the rest continued.’ (B11)

Almost all participants shared that there was nothing they dislike about the programme. They were satisfied with the programme arrangement. Participants appreciated the handy and informative resource book provided, and they could ‘refer to it when we have time and when we encounter any situations’ (B1). They liked the nutritious healthy snacks during breaktime, enjoyed listening to their peers’ health experiences, and learning from them. Two participants added that they appreciated the small group sessions (B1, B4). The home visits allowed them to ‘say innermost thoughts that I have never talked about’ (B9), ‘understand own self better’ (A16), and ‘enquire information if encounter personal issues’ (B7). Other participants fed back that the location of the programme was near their residence and was good.

Participants were satisfied with the instructor’s positive and lively class engagement. Few of them highlighted that the relationship with the instructor and ‘how the instructor explains, leads and drives the programme is important’ (A9).

‘The interaction with the current programme team is good. Relationship with instructor is important. If I do not like the instructor, I will not go.’ (A17)

##### Suitability for seniors

Participants reflected that the SHAPE intervention was a tailored programme closely aligned to the lives and needs of ‘every older person, not just limited to those living alone or with spouse only’ (A12). The participants fed back that the physical exercises introduced were ‘different from (other) outside classes’ (A12), challenging but doable and safe for older adults and most participants. Doing the exercises during group sessions ‘made the programme purposeful’ (A18). Few participants highlighted that not every participant can accept the exercises due to their medical conditions and the instructor modified the exercises to cater to participants’ physical needs.

‘Exercises are tailored according to participants' health conditions... Because the programme has a variety of exercises. Not every participant can accept. If they cannot accept (or do), they can sit at the side and watch or do modified exercise determined by instructors’ (B1)

Some participants voiced that the programme could be further refined to better cater to older adults’ specific needs and preferences. While homework provided was ‘not difficult and manageable’ (A9), some participants faced difficulty in expressing and writing their responses due to their limited language competencies. While some participants were satisfied with the programme timeslots, others found it inconvenient as they had to rush home to prepare/make dinner. Although participants appreciated the home visits, views towards the duration of home visits were mixed. While some fed back that the duration of home visits was acceptable, others preferred to have a single home visit, keeping it to 2 to 3 h.

### Results on outcome measures

Table 4 presents within-group changes in outcome measures among intervention and control group participants. Intervention participants reported significant increase in overall health-promoting behaviours (*P* < 0.001), health responsibility (*P* = 0.006), physical activity (*P* = 0.013), spiritual growth (*P* = 0.007) and stress management (*P* = 0.011). Although total SOC improved among intervention participants, the change was not significant. No changes were observed in SOC components, self-efficacy, self-rated health, total QoL and most of the QoL components among both intervention and control participants. However, intervention participants reported a significant drop in autonomy post-intervention (*P* = 0.04).

**Table 4:** Within-group changes in outcome measures among intervention and control group participants (*N* = 34)

**Outcome**	**Intervention group (*n* = 16)**			**Control group (*n* = 18)**		
	**Baseline**	**12-week**	*P*-value*	**Baseline**	**12-week**	*P*-value*
**SOC**						
Total score	64.19 ± 7.04	66.5 ± 9.24	0.29	66.50 ± 9.24	65.56 ± 10.85	0.98
Comprehensibility	24.13 ± 2.90	24.56 ± 3.60	0.62	25.39 ± 3.48	25.00 ± 4.80	0.70
Manageability	19.81 ± 3.19	20.63 ± 4.62	0.50	19.67 ± 3.82	20.17 ± 4.68	0.68
Meaningfulness	20.25 ± 3.19	21.31 ± 2.92	0.19	20.44 ± 2.38	20.39 ± 3.42	0.96
**Self-efficacy**	27.38 ± 6.13	28.13 ± 6.12	0.34	27.22 ± 4.22	28.11 ± 5.87	0.58
**QoL**						
Total score	91.06 ± 7.77	90.43 ± 8.94	0.61	88.11 ± 10.69	88.50 ± 10.45	0.82
Sensory Abilities	15.00 ± 3.35	15.44 ± 3.38	0.37	15.44 ± 2.68	14.83 ± 2.64	0.22
Autonomy	16.69 ± 2.36	15.75 ± 2.44	0.04	14.44 ± 2.66	15.00 ± 2.20	0.23
Past, Present, Future	15.13 ± 2.19	15.19 ± 2.01	0.90	14.33 ± 1.97	14.78 ± 2.10	0.43
Social Participation	15.75 ± 2.41	15.50 ± 1.97	0.56	15.78 ± 2.02	15.33 ± 1.68	0.24
Death & Dying	14.06 ± 3.13	14.00 ± 1.96	0.92	14.72 ± 2.87	14.22 ± 3.38	0.42
Intimacy	14.44 ± 2.97	14.56 ± 2.50	0.89	13.39 ± 3.26	14.33 ± 3.01	0.29
**HPLP**						
Total score	135.63 ± 20.08	147.88 ± 22.33	< 0.001	133.50 ± 14.33	134.22 ± 19.55	0.83
Health responsibility	22.00 ± 5.45	24.50 ± 5.20	0.006	21.11 ± 3.64	22.67 ± 4.31	0.08
Physical activity	17.81 ± 3.75	20.44 ± 4.21	0.013	19.28 ± 3.82	19.17 ± 4.44	0.90
Nutrition	26.44 ± 4.29	27.25 ± 4.23	0.250	26.17 ± 3.76	24.94 ± 4.78	0.22
Spiritual growth	24.19 ± 5.05	27.63 ± 5.48	0.007	22.94 ± 3.83	24.56 ± 3.47	0.09
Interpersonal relations	23.94 ± 4.54	25.19 ± 4.05	0.13	23.67 ± 2.63	22.61 ± 4.19	0.22
Stress management	21.25 ± 4.01	22.88 ± 4.81	0.011	20.33 ± 3.71	20.28 ± 3.69	0.94
**Self-rated health**	3.56 ± 1.09	3.81 ± 0.98	0.22	3.61 ± 0.70	3.50 ± 0.92	0.54
**Self-rated health (VAS)**	75.31 ± 15.76	78.44 ± 16.10	0.33	77.39 ± 10.31	76.40 ± 12.10	0.66

*SOC* Sense of Coherence, *QoL* Quality of life, *HPLP* Health Promoting Lifestyle Profile, *VAS* Visual Analogue Scale

^*^P-value for paired t-test

## Discussion

This pilot study evaluated the SHAPE intervention, a health resource programme, designed to equip older adults living alone or with spouses with better coping behaviours in maintaining health and independence at old age. The strengths of this study included the use of randomised controlled design and comprehensive assessment of implementation feasibility, intervention acceptability, and measurement of health-related outcomes. It yielded positive findings on most feasibility outcomes, intervention acceptability, and on health-promoting behaviours of older adults. The conduct of this pilot study was instrumental in identifying potential facilitators and barriers of implementing larger trials, which would be needed to confirm the effectiveness of SHAPE intervention.

### Feasibility of SHAPE intervention

Although multiple recruitment strategies were adopted, the recruitment rate of this pilot study fell below the established criteria (20%). Previous preventive lifestyle trials such as Lifestyle Matters and Food and Immunity studies reported lower recruitment rates [54, 55]. Although low recruitment rates were expectant of preventive trials compared to treatment trials [54, 56], it was a concern as community engagement efforts such as door-to-door canvassing were resource-intensive. However, successful recruitment efforts in this pilot trial were primarily based on interpersonal face-to-face recruitment, which were more effective than impersonal strategies such as fliers. With the rise in reported scam cases in Singapore, older adults who were approached were wary and guarded. Apart from the dedication of time and interpersonal skills of research personnel to build trust with the approached older adults [57, 58], perceived personal benefit was a strong motivation for older adults to participate in such health promotion research [59, 60]. Among the older adults who declined study participation, we observed that many cited reasons linked to personal beliefs or fears of engaging in social activities and learning at old age. Such views might suggest underlying issues which have an influence on reduced social participation at late life [61] and hinder seniors’ participation in learning activities [62, 63]. A recent systematic review reported that higher eligibility and enrolment rates were observed among studies which recruited older adults at risk of social isolation via recognised agencies (e.g., general practitioners, social services, community service providers) [64]. For more effective recruitment strategies in future trials, agency referral in combination with community engagement efforts could be adopted.

### Acceptability towards SHAPE intervention

Overall, participants were satisfied with the SHAPE programme and the intervention adherence was high. Most participants strongly agreed on the usefulness and relevancy of programme, citing that they benefited from the practical health knowledge on self-care and ageing and the programme content was tailored to all older adult adults, regardless of their living arrangement. This justified that SHAPE was a health resource programme that addressed the needs of older adults. The conduct of our prior qualitative study contributed aptly to the identification of healthy ageing stressors, which was critical in aligning with older adults’ demands of ageing experiences [65]. As participants perceived the SHAPE intervention to be applicable to all older adults, future larger trials need not exclude community-dwelling older adults with alternative living arrangements.

### Potential effects of SHAPE intervention

Intervention participants reported an increased in engagement of health-promoting lifestyle behaviours, particularly in health responsibility, physical activity, spiritual growth, and stress management. These quantitative findings were consistent with participants’ qualitative responses that they were more proactive and driven in taking actions for their health. Participants practiced the taught physical exercises, made changes to their dietary habits, and pursued greater social/leisure activities. As the SHAPE intervention equipped participants with the know-how in managing stressors of healthy ageing, participants could put these informational resources into practice. Additionally, the focus on positive ageing in SHAPE programme could also have improved participants’ expectations and attitudes towards ageing and spurred them to be active agents in health management [66, 67]. Although the SHAPE programme comprised several spiritual-related intervention activities, it was interesting to observe an improvement in spiritual growth among our intervention participants. These spiritual-related activities included personal reflections on meaning of health and life at old age, discussing existential issues such as preparing for end-of-life care and death, and the goal-setting exercises; The SHAPE intervention was secular. Intervention participants might have self-realisation beyond illness, perceived self with sense of wholeness and interconnectedness with the ageing assets in their environment [68]. Alternatively, the SHAPE programme might have enhanced participants’ self-care agency and thus increased their awareness towards the experienced contentment with self and life, positive self-concept, continuing personalisation, and spiritual growth [69].

The intervention participants in this pilot study who were younger in age reported a weaker SOC at baseline compared to the control participants. Past studies have shown that SOC could increase with age among older people [70–72]. Although the intervention participants reported an increase in total SOC, the change was not significant and minimal changes were observed in comprehensibility, manageability, and meaningfulness. Nonetheless, the intervention participants shared that the programme helped to straighten their thoughts towards their ageing experiences, such as having a more positive and coherent understanding towards life at old age and engaging in more healthful behaviours. However, our quantitative findings differed from a feasibility study on salutogenic self-care program which reported significant improvements in total SOC, comprehensibility, and manageability among older adults [73]. This could be due to a larger sample size of the latter’s randomised controlled pilot study. Literature has also shown that SOC can decrease due to negative life events such as health deficits, loss of independence and provision of spousal caregiving [74–76]. In the course of the SHAPE intervention, a few intervention participants shared that they experienced the loss of loved ones or a health event. However, encounters with negative life events were not measured in this pilot study. Future intervention studies could include measurements on negative life events to identify any possible confounders.

No significant changes were observed in self-rated health, total QoL and in most QoL components post-test among participants. The lack in statistical outcomes was likely due to our small sample size. However, intervention participants reported a significant small decline in autonomy. This finding contrasted with the qualitative responses that participants reported better control of their lives and it is unclear what contributed to the decrease in autonomy. Perhaps owning to the way how the items on WHOQoL-OLD were framed, participants receiving SHAPE intervention were encouraged to reflect deeply on their lives and they might have realised that they have not exercised their autonomy to their potential during their late lives. Our study found no changes in self-efficacy post-test scores among participants. Although participants from intervention and control group understood the items in GSE instrument, some of them enquired the context of each item. On hindsight, there might be a need to consider using a behaviour-specific self-efficacy instrument. According to Bandura [77], self-efficacy is task-specific and items in an ‘one measure fits all’ instrument using the GSE might be less sensitive for this study which included multi-domain lifestyle behaviours. It was observed that other elderly-related behavioural interventions measured specific self-efficacy tasks [78, 79].

### Other intervention considerations

Participants in this pilot study were recruited from the community based on their living arrangement and cognitive abilities. Their health and daily needs, literacy levels, and learning abilities were heterogeneous. As such, individual areas of improvements highlighted by the participants reflected differences in preferences, expectations of the program and their learning abilities. More timeslots could also be offered to participants. Facilitation of the present study, as well as future group sessions and home visits need to be adaptive and flexible yet adhering to the programme activities and learning outcomes stipulated in the intervention manual to ensure intervention fidelity when implementing this complex person-centred intervention [80]. For example, the assigned weekly homework which encouraged participants to recap and reflect on content learnt could adopt multiple choice responses or allow participants to draw to express themselves.

The conduct of this pilot trial was vital and useful in providing the research team with training and experiences to confirm and enhance competencies needed to conduct the main trial investigation and intervention [46]. Intervention participants highlighted that their positive interaction with the instructor/ resource facilitator contributed to their adherence of intervention and enjoyable learning experiences. The creation of positive social interactions with the intervention staff, which is dependent on the skill sets of the staff, could have provided social support and relatedness to participants, contributing to intervention adherence [81]. Proficiency in facilitation, establishing rapport and trust and the use of therapeutic communication skills were observed to be critical in harnessing benefits of group education and managing sensitive discussion topics such as death and dying among older people. Resource facilitators would need to constantly assess older adults’ understanding towards the discussion topics, engage them in an interesting and enthusiastic manner and be able to present complex information clearly using simple terms. For future larger trials, development and provision of train-the-trainer course might be needed to produce more resource facilitators.

SHAPE is essentially a resource-intensive intervention; it brings together different aspects of aging well, from accepting bodily changes, adopting healthy lifestyle habits, reconciling social role transitions, navigating complex healthcare system to preparing for end-of-life. Such approach of resource and knowledge integration with the health and wellness of community-dwelling older adults is critical as part of ageing populations’ health policy agenda on the shift from healthcare to health. Perhaps when scalability and effectiveness of the SHAPE intervention are conceivable, the intervention could be delivered at aged care community centres with trained healthcare professionals conducting the home visits. Prudent measures on developing core competencies of resource facilitators, refining the programme’s structure and operational conduct would be warranted. Sustainability of SHAPE would require detailed planning on the administrative support, investment of manpower training and fostering of a strong collaborative partnership with community service providers to achieve optimal engagement among the older adults, resource facilitators and community partners.

### Study limitations

There were several limitations to this study. As the intervention was piloted in Mandarin language, it excluded non-Mandarin speakers and the study sample consisted only Chinese older adults. No repeated measures were adopted to test the feasibility of multiple data collection time points for main trial. Blinding of group allocation among participants might have to be reconsidered for the main trial as participants could mingled and shared their group allocation with peers from control group in this single-centre community-based study. We adopted convenience sampling for this pilot study. Similar to a past local intervention study conducted on older community-dwellers [82], this study had lesser males than females as a result. Future trials could adopt gender-stratified sampling strategy. Alternatively, Patzelt, Heim [83] suggested gender-specific considerations should be noted by focusing performance-oriented exercise activities for males and holistic social-oriented activities for females to motivate older adults to participate in health programmes. As all participants continued to engage in activities in the community on their own as part of usual care, this study did not measure their engagement in new activities which could be a potential confounder in determining the potential effects of the intervention.

## Conclusion

The SHAPE intervention was grounded by strong theoretical underpinning of the salutogenic model of health. Findings of this pilot trial were positive and supported that with protocol modifications, SHAPE can be a feasible and beneficial health resource intervention for older adults. The conduct of this pilot trial was essential and fruitful. It was a preview of future larger trial for the research team to gain training and experience, and to understand the demands and resources needed for larger trial implementation. Further modifications on recruitment strategies, eligibility criteria, selection of outcome measures, training of resource facilitators and strong collaboration bonds with community partners would be needed to increase feasibility robustness and scientific rigor of this complex intervention.

## Data Availability

The datasets generated during and/or analysed during the current study are not publicly available due to confidentiality and anonymity of participants. However data are available from the corresponding author upon reasonable request.

## Abbreviations

GDS: Geriatric Depression Scale
GRR: Generalised resistance resources
GSE: Generalized Self-efficacy Scale
HPLB: Health-promoting lifestyle behaviours
HPLP-II: Health Promoting Lifestyle Profile-II
MoCA: Montreal Cognitive Assessment
QoL: Quality of Life
SHAPE: Salutogenic Healthy Aging Programme Embracement
SOC: Sense of coherence
WHOQoL-OLD: World Health Organisation Quality of life Old module
